# Differences in the Structure and Function of the Vestibular Efferent System Among Vertebrates

**DOI:** 10.3389/fnins.2021.684800

**Published:** 2021-06-23

**Authors:** Kathleen E. Cullen, Rui-Han Wei

**Affiliations:** ^1^Department of Biomedical Engineering, Johns Hopkins University, Baltimore, MD, United States; ^2^Department of Otolaryngology, Johns Hopkins University School of Medicine, Baltimore, MD, United States; ^3^Department of Neuroscience, Johns Hopkins University, Baltimore, MD, United States; ^4^Kavli Neuroscience Discovery Institute, Johns Hopkins University, Baltimore, MD, United States

**Keywords:** vestibular, neural coding, multimodal, visual, somatosensory, efference copy, perception, evolution

## Abstract

The role of the mammalian vestibular efferent system in everyday life has been a long-standing mystery. In contrast to what has been reported in lower vertebrate classes, the mammalian vestibular efferent system does not appear to relay inputs from other sensory modalities to the vestibular periphery. Furthermore, to date, the available evidence indicates that the mammalian vestibular efferent system does not relay motor-related signals to the vestibular periphery to modulate sensory coding of the voluntary self-motion generated during natural behaviors. Indeed, our recent neurophysiological studies have provided insight into how the peripheral vestibular system transmits head movement-related information to the brain in a context independent manner. The integration of vestibular and extra-vestibular information instead only occurs at next stage of the mammalian vestibular system, at the level of the vestibular nuclei. The question thus arises: what is the physiological role of the vestibular efferent system in mammals? We suggest that the mammalian vestibular efferent system does not play a significant role in short-term modulation of afferent coding, but instead plays a vital role over a longer time course, for example in calibrating and protecting the functional efficacy of vestibular circuits during development and aging in a role analogous the auditory efferent system.

## Introduction

**FIGURE 1:**
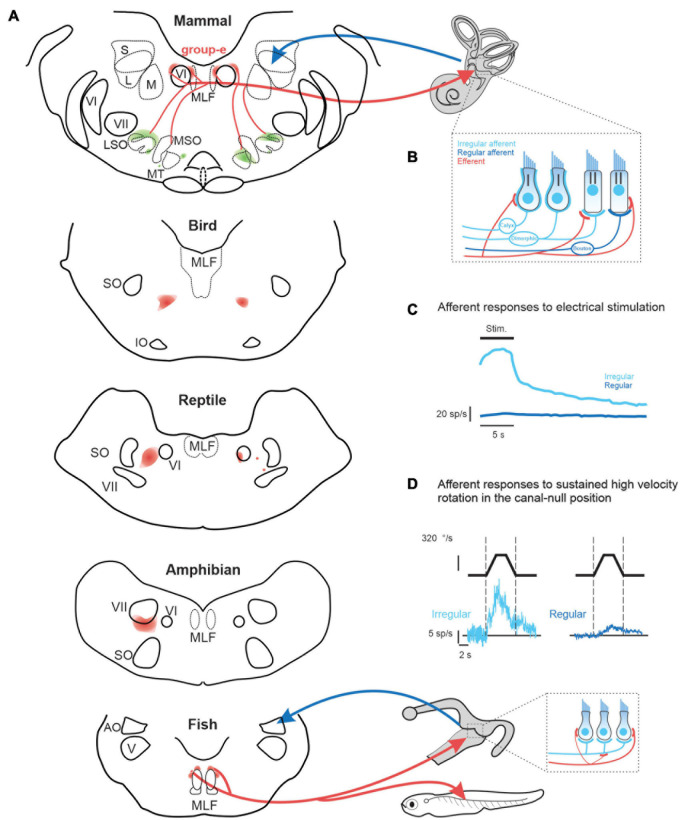
**(A)** of vestibular efferent (red) and afferent (blue) projections across vertebrates including: mammals (squirrel monkey: Goldberg and Fernandez, 1980), birds (pigeon: Eden and Correia, 1981), reptile (lizard: Barbas-Henry and Lohman, 1988), amphibians (toad: Pellegrini et al., 1985), and fish (toadfish: Highstein and Baker, 1986). **(B)** In mammals, irregular afferents (light blue) typically innervate both type I and II hair cells (i.e., dimorphic afferents), while regular afferent (dark blue) more selectively innervate type II hair cells. Note that the mammalian vestibular efferent system innervates type II hair cells and their afferent bouton endings, as well as the afferent calyces of type I hair cells (Lysakowski and Goldberg, 1997, 2008). In contrast, in lower vertebrates, which do not have hair cells with calyx endings, the vestibular efferent system innervates type II hair cells and, in some species, such as the toadfish (Sans and Highstein, 1984) also their afferent bouton endings [*bottom inset*, **(A)**]. **(C)** Comparison of the population-averaged efferent-mediated responses of irregular vs. regular afferents to electrical microstimulation of central “group-e” neurons (Goldberg and Fernandez, 1980). **(D)** Efferent-mediated population responses of irregular (*left*) vs. regular (*right*) afferents to sustained high amplitude rotation in the canal-null position (Sadeghi et al., 2009). Note, in contrast to their conventional evoked by natural head motion, afferents displayed excitatory responses for stimulation in both directions—termed a type III response. AO, nucleus anterior octavus; LSO, MSO, MT, lateral and medial superior olive and medial nucleus of the trapezoid body; MLF, medial longitudinal fasciculus; IO, inferior olive; S, L, M, superior, lateral and medial superior vestibular nucleus; SO, superior olive; V, trigeminal nucleus; VI, abducens nucleus; VII, facial nucleus.

While the function of the mammalian auditory efferent system is well understood, the role of the mammalian vestibular efferent system remains a mystery. Yet the peripheral vestibular system (i.e., the sensory epithelium of the three semicircular canals and two otoliths) receives central projections from the vestibular efferent system in all vertebrate species (Meredith, 1988; Goldberg et al., 2012). Interestingly, there is considerable heterogeneity in the organization and location of the efferent cell bodies across different vertebrate classes (Figure 1A). In four of the five classes of vertebrates –fish, amphibians, reptiles, and birds—the efferent neurons that project to the vestibular and auditory periphery are localized in a single cell group. In aquatic vertebrates, efferent neurons within this cell group also innervate lateral line neuromasts. In such vertebrates, a single efferent neuron can innervate the sensory epithelium of both the vestibular and lateral-line systems (larval Xenopus: Claas et al., 1981; fish: Highstein and Baker, 1986; Meredith and Roberts, 1987). In contrast to the other four vertebrate classes, the organization of the vestibular efferent system is markedly different in mammals as compared. Notably, the cell bodies of efferents innervating the mammalian vestibular and auditory periphery are located in separate nuclei; the vestibular efferent nucleus is commonly referred to as the “group-e,” whereas the auditory efferent nucleus is the superior olivary complex.

In this review, we consider differences in both the structure and function of the vestibular efferent system across vertebrate classes. We focus on how the vestibular efferent systems transmit extra-vestibular sensory and motor information to the vestibular periphery in lower vertebrates (i.e., fish and amphibians). We also discuss how this extra-vestibular information influences the responses of vestibular afferents in these aquatic vertebrates. Additionally, we contrast these findings with those of studies in mammals establishing the absence of such efferent-mediated effects. Taken together, this evidence suggests an evolution in the primary role of the vestibular efferent system. We speculate that instead of playing a key role in the short-term modulation of afferent coding as it does in aquatic vertebrates, the function of the mammalian vestibular efferent system is to regulate pathway efficacy during development in a role analogous the auditory efferent system.

### The Vestibular Efferent System: Organization and Embryologic Origin

Vestibular, auditory, as well as lateral line efferents are thought to have a common embryologic origin that is shared with facial and branchiomotor motoneurons (Fritzsch, 1996). Accordingly, both vestibular and auditory efferent neurons in some lower vertebrates (e.g., eels and toads) are located within or overlapping the facial nucleus. However, there is considerable heterogeneity in the specific location of vestibular efferent neurons both across vertebrate classes and across different mammalian species. For example, in reptiles and birds the efferent nucleus is distinct from the facial nucleus, and is located more dorsally (Figure 1A, *bottom*). While a single nucleus comprises both auditory and vestibular efferents in these vertebrates, the somas of vestibular efferents tend to be located more dorsally (reviewed in Goldberg and Cullen, 2011). In contrast, the cell bodies of the mammalian vestibular efferent system are located in the group-e nucleus, which is distinct from the auditory efferent system. The mammalian group-e nucleus is located even further from the facial nucleus, with the vestibular efferent somas situated dorsal to the genu of the facial nerve and just medial to the VI (abducens) nucleus (Figure 1A, *top*). There is likewise heterogeneity in the dendritic morphology of the vestibular efferent system across vertebrate classes. The dendrites of efferent neurons in fish and amphibians span a large portion of the brainstem, whereas the dendrites of group-e neurons in mammals span a far more limited area and are relatively sparse. These striking differences in the anatomy and morphology of the vestibular efferent system likely underlie differences in its functional role across vertebrate classes (detailed further below).

### The Vestibular Efferent System in Mammals: A Neural Circuit for the Modulation of Motion Sensing

Across species the mammalian vestibular efferent system comprises only about 300 neurons on each side and sends bilateral projections to the peripheral vestibular system (Goldberg and Fernandez, 1980; Marco et al., 1993). The projections of an individual efferent axon can be profuse, spanning multiple vestibular organs (Purcell and Perachio, 1997), where they target type II vestibular hair cells as well as the afferents innervating both type I and II hair cells (Figure 1B, reviewed in Goldberg, 2000). Experiments in monkey, cat, and chinchilla have shown that electrical microstimulation of the mammalian “group-e” nucleus (Figure 1A) evokes comparable excitatory responses in the vestibular afferents on both sides (Figure 1C; Goldberg and Fernandez, 1980; Marlinski et al., 2004; McCue and Guinan, 1994). However, there are important differences in the magnitude of efferent-activated responses across individual afferents. In particular, the responses evoked in afferents with more “irregular” resting discharges are an order of magnitude greater than those evoked in their regular counterparts (Figure 1C).

Importantly, both canal and otolith afferent fibers can be classified based on the regularity of their resting discharge. In general, regular afferents preferentially provide bouton endings to type II hair cells, whereas irregular afferents have larger axons and either transmit information from the type I hair cells located at the center of neuroepithelium or integrate inputs from both type I and II hair cells. The firing rates of regular afferents in turn encode more information about head motion than irregular afferents (Sadeghi et al., 2007). On the other hand, more recent studies (Jamali et al., 2016, 2019) have shown that irregular afferents better discriminate between head motion stimuli through differential patterns of precise (∼6 ms) spike timing than regular afferents. These two parallel streams of sensory input provided by regular and irregular afferents are preserved and further refined in mammalian central vestibular pathways. Notably, regular and irregular afferents preferentially target vestibulo-ocular reflex (VOR) and vestibulo-spinal pathways, respectively (reviewed in Cullen, 2019). Thus, the fact that efferent-activated responses are greater for irregular than regular afferents suggests that the mammalian vestibular efferent system could potentially play a more significant role modulating vestibulo-spinal vs. vestibulo-ocular reflex) pathways.

To date, however, no study has directly recorded the responses of mammalian vestibular efferent neurons. The sparsity of the target population presents a challenge for definitively identifying efferent units. Early experiments in lower vertebrates (fish and amphibians) have established that the vestibular efferent system integrates information across multiple vestibular end organs and from both labyrinths (Gleisner and Henriksson, 1963; Schmidt, 1963; Precht et al., 1971; Blanks and Precht, 1976; Hartmann and Klinke, 1980). Vestibular efferents target the vestibular periphery, and do not make synaptic contacts with neurons within the vestibular nuclei (reviewed in Holt et al., 2011). As such the vestibular efferent system can only modulate the afferent input to vestibular nucleus neurons by inducing significant changes in afferent responses (e.g., in contrast to the presynaptic control observed in dorsal root ganglion). Thus, subsequent studies in mammals have provided insight into the responses of vestibular efferent neurons by recording from individual vestibular afferents.

Recordings made from semicircular canal afferents during have high velocity rotations have further demonstrated that the efferent vestibular circuitry is functional in mammals. These studies used a sophisticated experimental design in which the conventional semicircular canal afferent responses to head rotation were first minimized by positioning the head in the null plane for the superior semicircular canal (Plotnik et al., 2002, 2005; Sadeghi et al., 2009). With the head in this null orientation, sustained steps of high constant-velocity rotation (> 300 °/s) were then passively applied. In response, superior semicircular canal afferents displayed excitatory responses for both rotation directions—termed a type III response. The vestibular source of the efferent-mediated responses was then conformed by plugging the horizontal and posterior canals (Sadeghi et al., 2009). Importantly, these type III responses markedly contrast with the characteristic type I responses of afferents evoked by natural head rotations, for which rotations in the opposite directions lead to excitatory vs. inhibitory responses (Figure 1D). Furthermore, type III responses evoked in irregular afferents were an order of magnitude greater than those evoked in their regular counterparts (Figure 1D). Moreover, this high velocity rotation in the null plane (Figure 1D), similar to microsimulation of group-e neurons (Figure 1D) evoked responses in irregular afferents with comparable dynamics, which notably included a slow response component with a long time constant of 5–20 s (Goldberg and Fernandez, 1980; Sadeghi et al., 2009). Thus, artificial activation of the vestibular efferent system via both microstimulation and sustained high velocity rotation appears to predominately alter coding in irregular afferents and, in turn, the time course of this activation is relatively long relative to the head movements experienced during natural self-motion (Carriot et al., 2014, 2017).

### Synaptic Physiology of the Mammalian Vestibular Efferent System: Implications for Function

Acetylcholine (ACh) is the main neurotransmitter at the vestibular efferent synapses across vertebrate classes (reviewed in Goldberg et al., 2012). Furthermore, pharmacology of the vestibular efferent synapses has additional complexity. First, the neuroactive peptide calcitonin gene-related peptide (CGRP) is widely present in the vestibular efferent neurons of mammals, fish and amphibians (Sewell and Starr, 1991; Highstein, 1992; Eybalin, 1993; Bailey and Sewell, 2000) and is co-expressed by vestibular efferent neurons and peripheral efferent terminals (Ohno et al., 1991; Luebke et al., 2014). Second, additional substances are commonly co-expressed in cholinergic neurons, including, adenosine 5′-triphosphate (ATP), dopamine (DA), GABA, and neuronal nitric oxide synthase (nNOS) have also been reported within vestibular efferent neuron terminals of the vestibular end organs (reviewed in Mathews et al., 2017).

Recent experiments in mammals (Schneider et al., 2021) have shown that muscarinic and nicotinic AChR antagonists block the slow and fast component of the excitatory responses induced in afferents by efferent microstimulation (i.e., Figure 1C). Furthermore, because the expression of CGRP accompanies development of the vestibular efferent system, it has been proposed that the vestibular efferent system plays a slow modulatory role in shaping the functional connectivity/efficacy of the peripheral organs during maturation (Holt et al., 2011). Consistent with this idea, CGRP null mice demonstrate a substantial reduction in the efficacy of their vestibulo-ocular refex (Luebke et al., 2014). Interestingly, as noted above, electrical microsimulation of mammalian vestibular efferent neurons generates an increase in vestibular afferent activity (see review, Holt et al., 2011). In contrast, electrical microstimulation of the auditory efferent system suppresses auditory afferent nerve activity. Given that ACh is the primary neurotransmitter released by auditory and vestibular efferent neurons and hyperpolarizes the hair cells in both sensory systems, the difference in excitation vs. inhibition of vestibular vs. auditory afferents initially difficult to reconcile. However, recent studies have established the synaptic mechanisms by which efferent-mediated hyperpolarization vestibular hair cells leads to excitation of vestibular afferent activity in mammals (reviewed in Poppi et al., 2020).

### Extra-Vestibular Sensory Integration in the Vestibular Periphery: Strategies Differ Across Vertebrate Classes

The discovery that (i) microstimulation or (ii) sustained high velocity vestibular stimulation alters the responses of vestibular afferents indicates that the vestibular efferent system circuitry remains functional in mammals. This has led to the common view that the efferent neurons may modulate the activity of vestibular afferents in more natural conditions as well. However, it is important to emphasize that electrical and rotational stimuli used in these two experimental approaches are unnatural and thus not actually experienced in everyday life. Indeed, it had proven difficult to find more natural circumstances that lead to large efferent-mediated responses in the mammalian vestibular nerve, even in irregular units. Further, it must be recognized that even the responses sustained high velocity vestibular are small (≈10 spikes/s) compared with those produced by conventional afferent stimulation (>200 spikes/s) to the same stimuli (Sadeghi et al., 2007). To date, no study has directly characterized the responses of mammalian vestibular efferent neurons during natural stimulation. Instead, prior experiments have only reported the responses of vestibular efferents in lower vertebrate classes, namely fish and amphibians. Studies in these lower vertebrates have established that individual vestibular efferents respond to extra-vestibular sensory stimulation, thus providing a substrate for the integration of vestibular and extra-vestibular inputs at the level of the vestibular periphery.

In particular, vestibular efferent neurons in fish and amphibians respond to somatosensory inputs produced by passively manipulating the limbs and applying pressure to the skin (goldfish: Hartmann and Klinke, 1980; toadfish: Highstein and Baker, 1985; frog: Schmidt, 1963; salamander: Schmidt, 1965). Additionally, there are reports that vestibular efferents can be driven by a visual or auditory stimulation in these two classes of vertebrate (visual—goldfish: Schmidt et al., 1972; Hartmann and Klinke, 1980; frog: Caston and Bricout-Berthout, 1982; auditory—toadfish: Highstein and Baker, 1985; frog: Bricout-Berthout and Caston, 1982). The above findings in fish and amphibians raise two fundamental questions. First, how do the vestibular and extra-vestibular sensory signals carried by vestibular efferent neurons modify the responses of the **vestibular afferents** that they target in fish and amphibians? Second, is this strategy conserved across all vertebrate classes—including mammals?

Indeed, with respect to the first question, the observation that activation of the “vestibular system” can produce efferent-mediated responses in **vestibular afferents** appears to be common across vertebrates. However, the sign and magnitude of this effect does vary. For example, a neurophysiological study in birds demonstrated heterogeneity in efferent-mediated afferent responses evoked by stimulation of the horizontal semicircular canal on the contralateral side (Dickman and Correia, 1993). Both excitation and inhibition were observed across individual afferents. In contrast, as reviewed above, stimulation of the vestibular system in mammals always produces excitatory efferent-mediated responses in vestibular afferents. Specifically, high amplitude vestibular stimulation (∼300 deg/s) evoked an increase in afferent firing rate regardless of movement direction in both anesthetized chinchilla and alert rhesus monkey (Figure 1C; Plotnik et al., 2002; Sadeghi et al., 2009). However, it is notable that these effects were relatively less marked in alert monkeys.

**FIGURE 2:**
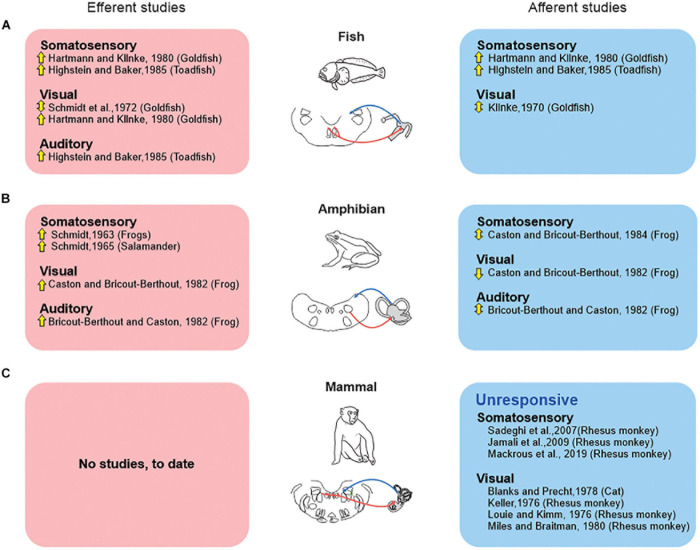
Extra-vestibular sensory systems (somatosensory, visual, auditory) have been reported to alter peripheral vestibular processing in fish and amphibians but not in mammals. Studies that directly recorded from the vestibular efferent pathway (red boxes), vs. those that recorded efferent-mediated effects in vestibular afferents (blue boxes) are shown for studies in fish **(A)**, amphibians **(B)**, and mammals **(C)**. *Symbols*: up arrows: excitation; down arrows: inhibition; up-down arrows: both excitation and inhibition.

However, there are marked differences across species regarding the influence of “extra-vestibular” sensory input (i.e., somatosensory, visual, and auditory) on the responses of vestibular afferents. These differences indicate distinct strategies among vertebrates (Figure 2). Classic studies in fish (Figure 2A) demonstrated that somatosensory stimulation activates individual vestibular afferents (Hartmann and Klinke, 1980; Highstein and Baker, 1985), and that visual stimulation can evoke directionally sensitive responses (Klinke, 1970). Likewise, somatosensory and visual stimulation has also been reported to alter the firing activity of individual vestibular afferents in amphibians (Figure 2B; frog: Caston and Bricout-Berthout, 1982, 1984, respectively). Yet, in this latter vertebrate class, somatosensory stimulation can both inhibit and excite afferents, and thus contrasts with what is seen in fish. Likewise, auditory stimulation can evoke either excitatory or inhibitory responses in individual afferents in amphibians (Figure 2B; Bricout-Berthout and Caston, 1982). Thus, in answer to the first question, it is clear that both the vestibular and extra-vestibular sensory signals carried by vestibular efferent neurons modify the responses of the **vestibular afferents** that they target in fish and amphibians and that this resultant modulation is generally excitatory in fish and excitatory or bidirectional in amphibians (see Figure 2).

This then leads to the second question of whether this strategy is conserved across all vertebrate classes—including mammal. The evidence to date indicates that the integration of vestibular and extra-vestibular sensory signals at the level of the vestibular periphery is not a strategy that is conserved across all vertebrate classes—including mammals. Importantly, and in contrast to the above findings in fish and amphibians, experiments in rhesus monkeys (Sadeghi et al., 2007; Jamali et al., 2009; Mackrous et al., 2019) have revealed that afferents are unresponsive to related to somatosensory/proprioceptive signals (Figure 2C). Specifically, afferent responses are unresponsive to proprioceptive stimulation alone, and respond identically to passive whole body (i.e., vestibular-only stimulation) and passive head-on-body movements (i.e., vestibular + proprioceptive stimulation). Likewise, visual stimulation does not alter primate vestibular afferent activity (Figure 2C). Vestibular afferents are further unresponsive to full-field visual motion in both cats (Blanks and Precht, 1978) and rhesus monkeys (Keller, 1976; Louie and Kimm, 1976; Miles and Braitman, 1980). Thus, to date, there is no evidence to support the proposal that extra-vestibular sensory stimulation in mammals can alter the responses of vestibular afferents.

### Behaviorally/Context Independent Coding in the Vestibular Periphery of Mammals

**FIGURE 3:**
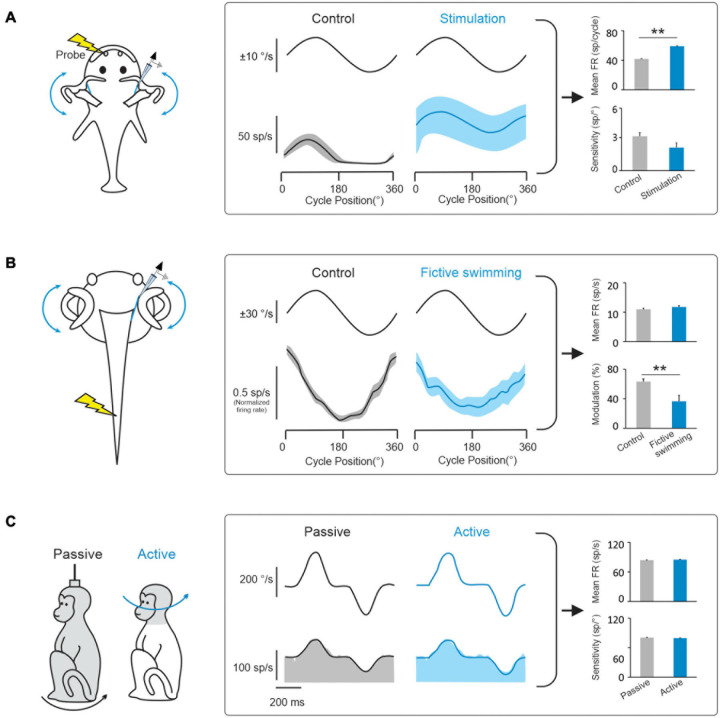
Behaviorally-dependent efferent-mediated responses have been reported in fish and amphibians but not in mammals. **(A)** *Left:* Inducing a behavioral escape response in a head fixed toadfish activates vestibular efferent neurons. *Middle:* Behavioral activation of efferents alters vestibular afferent responses to passive whole-body rotation in the head-restrained fish. *Right:* bar plots comparing effects of electrical efferent stimulation on background and rotation-induced firing of horizontal canal afferents (data replotted from Boyle and Highstein, 1990). **(B)** *Left:* Afferent recordings made in a semi-isolated *in vitro* anaesthetized larval Xenopus preparation, during passive whole-body rotation. Responses to this passive rotation were compared before and during bouts of fictive swimming induced via applied electrical stimulation. *Middle:* The induction of fictive swimming alters vestibular afferent responses. *Right*: Bar plots compare the effects of fictive swimming on background and rotation-induced discharges of horizontal canal afferents (data replotted from Chagnaud et al., 2015). **(C)** *Left:* Afferent recordings made in rhesus monkeys during active and comparable passive head motion. *Middle:* Primate afferent responses are the same during active and comparable passive head motion (reviewed in Cullen, 2019). *Right*: Bar plots compare afferents resting rates and sensitivities in these two conditions (Cullen and Minor, 2002). Note: error bars represent SEM; **P ≤ 0.001 (Wilcoxon signed-rank test).

During natural activities, vestibular information is integrated with motor signals, as well as other sensory signals (including visual and proprioceptive signals). In this context, a prevailing hypothesis has been that a key function of the vestibular efferent system is to alter peripheral motion sensing during active movements. In this view, the vestibular efferent pathway transmits motor-related signals to the vestibular periphery that modulate the responses of receptor cells within the semicircular canal and otolith sensory organs to effectively extend their head motion coding range during voluntary behaviors (reviewed in Goldberg, 2000; Mathews et al., 2017). Indeed, there is evidence for this proposal from studies of non-mammalian species (fish and tadpoles). Vestibular afferents in head-restrained toadfish display an increase in mean firing rate and a reduction in sensitivity to passive vestibular stimulation when preparing an escape response, which is thought to make them less likely to demonstrate inhibitory cut-off and/or saturation (Figure 3A; Boyle and Highstein, 1990; Rabbitt et al., 1995). Similarly, using a semi-isolated *in vitro* anaesthetized larval Xenopus preparation, showed that motor signals originating in the spinal locomotor circuitry are conveyed to the vestibular periphery to produce an overall reduction in afferent sensitivity to passive vestibular stimulation (Figure 3B; Chagnaud et al., 2015). The results of these studies have led to the common view that motor efference copies (i.e., an internal copy of the motor command) used to generate active behaviors are conveyed by the vestibular efferent system to the periphery to modulate responses in receptor cells and vestibular afferent fibers.

However, while studies in non-mammalian species provide support for the idea that the vestibular efferent system modulates peripheral sensory processing during active behaviors, this strategy does not appear to be common among all species. In particular, there is no direct evidence to date that the vestibular efferent system modulates afferent responses during active motion in mammals including primates. In rhesus monkeys, neither semicircular canal nor otolith afferent responses are altered during active orienting head movements or the generation of motor commands that activate the neck musculature (Figure 3C; Cullen and Minor, 2002; Sadeghi et al., 2007; Jamali et al., 2009; Mackrous et al., 2019). Furthermore, the motor pathways that control eye movements also do not alter the responses of vestibular afferents; the responses of semicircular canal and otolith afferents are insensitive to saccadic eye movements, smooth pursuit, and optokinetic nystagmus (Keller, 1976; Louie and Kimm, 1976; Miles and Braitman, 1980; Cullen and Minor, 2002; Sadeghi et al., 2007; Jamali et al., 2009). Finally, findings that afferents respond similarly in anesthetized and alert monkeys (Fernandez and Goldberg, 1971; Keller, 1976; Lisberger and Pavelko, 1986) and that afferent responses remain unchanged when animals are engaged in a vestibular heading discrimination task in which monkeys made saccades to indicate whether their perceived direction of translation was leftward or rightward relative to straight ahead (Yu et al., 2015). Thus to date, the evidence available indicates that the vestibular efferent system does not alter sensory processing by relaying behaviorally-dependent (e.g., motor commands that drive active head or eye movements) or context-dependent (e.g., alertness, feature attention) signals to vestibular periphery.

It is noteworthy that the above experiments, which found no change in in vestibular afferent responses during active orienting head movements in rhesus monkeys, included head movements that extended well into the amplitude range of other common active head movements, including those generated during locomotion (Carriot et al., 2017). Yet it is also important to note that these head movements are generated by the descending pathways to the neck musculature, rather than by the locomotor circuitry. This then raises the question of whether locomotion might preferentially alter the responses of afferents, via the vestibular efferent system, in mammals. Across species, locomotion provides us with the ability to explore our world and is critical for survival. The influential studies in aquatic vertebrates, described above (i.e., toadfish: Boyle and Highstein, 1990; larval xenopus: Chagnaud et al., 2015), are widely considered as support the idea that locomotion alters the responses of afferents via the vestibular efferent system. Importantly, however, neither of these studies actually recorded the afferent activity during voluntary active movement. Instead, the animals were head restrained and vestibular stimuli were passively applied. Accordingly, the question of whether the vestibular efferent pathway modulates peripheral coding of active head motion during locomotion remains open. We predict that it will be unlikely that such a strategy is utilized by higher vertebrates—notably primates. Instead, we speculate that coding of head motion by the vestibular periphery is context independent during natural active behaviors. This alternate strategy allows pathway-selective modulation of relationships between motor signals and the resultant vestibular feedback during active behaviors.

## Future Directions and Conclusion

The function of the mammalian vestibular efferent system in everyday life remains poorly understood. The finding that it can be artificially activated by electrical stimulation and/or high velocity vestibular stimulation (Figure 1) demonstrates that its circuitry is intact and operational in mammals, including chinchillas and monkeys. However, in contrast to what has been reported in lower vertebrate classes, the mammalian vestibular efferent system does not relay inputs from other sensory modalities to the vestibular periphery (Figure 2). Instead, the integration of vestibular and extra-vestibular information occurs only at next stage of processing in the mammalian vestibular system—specifically, at the level of the first central neurons within the vestibular nuclei. For example, VOR pathways integrate gaze commands that suppress the VOR when the behavioral goal is to voluntarily redirect gaze (Roy and Cullen, 1998, 2002). Further, vestibulo-spinal and thalamocortical pathways integrate proprioceptive and voluntary head movement commands that suppress these pathways during voluntarily motion relative to space (Roy and Cullen, 2004; Brooks and Cullen, 2014; Dale and Cullen, 2019). Additionally, in further contrast to what has been observed in lower vertebrate classes, the mammalian vestibular efferent system does not function to prevent overstimulation by the voluntary movements experienced during everyday behaviors such as active head movements and locomotion (Figure 3; Cullen and Minor, 2002; Sadeghi et al., 2007; Jamali et al., 2009).

What then is the physiological role of this system in mammals? One possibility is that mammalian vestibular efferent system does not play a role in short-term modulation of afferent coding, but instead plays a role in modifying sensory encoding over a longer time course. In this regard, our knowledge of the function role of the auditory efferent system may provide some clues, given that the vestibular and auditory systems have a common phylogenetic origin (reviewed in Fritzsch, 1992). The auditory efferent system can induce long-term plastic changes in afferent physiology following cochlear de-efferentation (Liberman, 1990). Thus, one possibility is that the vestibular efferent system similarly plays a role in the similar role in the plasticity of the vestibular system following a similar perturbation, such as the loss of peripheral vestibular nerve input. Surprisingly, however, an experiment testing whether the vestibular efferent system plays a role in rebalancing input from the two labyrinths, after compensation to lesion of vestibular nerve on one side, found no change in afferent mean resting rates or sensitivities recorded in the *intact* nerve on the other side (i.e., the contralesional nerve; Sadeghi et al., 2007). Likewise, although the vestibular system shows remarkable plasticity in response to the altered environmental requirements induced by the wearing of visual lenses, the head velocity sensitivity or resting discharge of monkey vestibular afferents are unchanged following weeks of such visually induced plasticity (Miles and Braitman, 1980). Interestingly, in the above compensation study, Sadeghi et al. (2007) did report a small but significant increase in the proportion of irregular afferents and a decrease in the proportion of regular afferents. This result fits with the recent finding that inactivation of vestibular efferent fibers in mice preferentially influences the firing of irregular afferents (Raghu et al., 2019), and could provide insight into the role of the mammalian efferent system.

Another possibility is that a primary function of the mammalian vestibular efferent system is to calibrate pathway efficacy during development– in a role analogous to that shown for auditory efferents (Walsh et al., 1998; Lauer and May, 2011; Mishra, 2020). Indeed, as reviewed above there are some notable parallels between the synaptic physiology of the auditory and vestibular efferent pathways. More specifically, the auditory efferent system comprises the medial (MOC) and lateral olivocochlear (LOC) systems. The mechanism of synaptic transmission in the MOC pathway is similar to that in the vestibular efferent system; activation of both systems results in acetylcholine releases, which in turn hyperpolarizes receptor cells in the periphery via activation of α9/α10 nAChRs (Ballestero et al., 2011). Nevertheless, the vestibular efferent system also shares a similarity with the LOC system, namely that the neuroactive peptide calcitonin-gene related peptide (CGRP) acts at efferent synapses and their targets in both systems (Wackym et al., 1993; Maison et al., 2003; Ahn et al., 2009; Xiaocheng et al., 2013). Importantly, the efferent innervation of the cochlea occurs early in development (reviewed in Simmons, 2002). In particular, medial efferent neurons mature first and initially project to the inner hair cell region of the cochlea and then synapse on outer hair cells, while lateral efferent neurons mature later and predominately project to the inner hair cell region. Accordingly, it has been proposed that the early efferent innervation of the cochlea plays an important role in shaping the functional connectivity/efficacy of peripheral auditory signaling. Likewise, the efferent innervation of the vestibular sensory organs occurs early in development. Maturing type I and type II hair cells initially receive direct efferent contacts, which in the case of type I hair cells are displaced during development by the calyx afferent terminals (Favre and Sans, 1979) to the outer face of calyx terminal. The use of transgenic mouse models with a targeted deletion loss of CGRP *from birth* has revealed impaired functional efficacy, specifically an attenuation of auditory nerve responses (Walsh et al., 1998; Lauer and May, 2011) and, as noted above, also a marked reduction in VOR gain (Luebke et al., 2014). Thus, disrupting both the auditory LOC and vestibular efferent pathways from birth causes deficits that are consistent with the view that both efferent pathways play a vital role in calibrating pathway efficacy during development. Furthermore, lesions of the mammalian auditory efferent pathway accelerate age-related hearing loss (Liberman et al., 2014), and reduces protection against loud noise exposure (reviewed in Lauer et al., 2021). Thus, we further speculate that lesions to the mammalian vestibular efferent pathway in mammals would accelerate age-related peripheral vestibular impairment (Merchant et al., 2000; Lopez et al., 2005) and reduce protection against noise-induced damage (reviewed in Stewart et al., 2020).

## Summary

Overall, the evidence available to date contradicts the common wisdom that the mammalian vestibular efferent system dynamically modulates sensory coding by the vestibular periphery during natural behaviors. Specifically, neurophysiological studies have demonstrated that the mammalian vestibular efferent system does not play a significant role in short-term modulation of vestibular afferent responses. Instead, the evidence to date suggests that the mammalian vestibular afferents encode head motion in a context-independent manner during natural behaviors, refuting an increasingly popular idea that efferent-mediated modulation of the vestibular periphery enhances postural and gaze stability during active behaviors (e.g., Jahn et al., 2000; Dietrich and Wuehr, 2019; Dietrich et al., 2020). Overall, the available evidence supports a competing hypothesis, namely that the mammalian efferent vestibular system predominantly plays a role over a longer time course, for example in calibrating vestibular pathway efficacy during neural development and/or protecting peripheral transmission during aging.

## Author Contributions

KC wrote the initial version of the manuscript with support from R-HW. R-HW provided critical feedback and helped shape the research arguments presented in the manuscript. Both authors contributed to the article and approved the submitted version.

## Conflict of Interest

The authors declare that the research was conducted in the absence of any commercial or financial relationships that could be construed as a potential conflict of interest.
